# Odontoid Process and Femur: A Novel Bond in Anatomy

**DOI:** 10.7759/cureus.7372

**Published:** 2020-03-23

**Authors:** Anastasios Vasilopoulos, Gregory Tsoucalas, Eleni Panagouli, Gregory Trypsianis, Vasilios Thomaidis, Aliki Fiska

**Affiliations:** 1 Anatomy, School of Medicine-Democritus University of Thrace, Alexandroupolis, GRC; 2 Anatomy, National and Kapodistrian University of Athens, Athens, GRC; 3 Biostatistics, School of Medicine-Democritus University of Thrace, Alexandroupolis, GRC

**Keywords:** long bone measurements, femur maximum length, anatomy, anthropology, forensics

## Abstract

Objective

The morphology and quantitative anatomy of the axis vertebra (C2) attracts a lot of attention between anatomists, surgeons and radiologists. However, no report exists in the literature correlating the height of the dens with the length of the femur. Our paper aims to determine such a correlation.

Material and methods

An examination of forty-five adult dry skeletons (twenty-three male and twenty-two female) was conducted. The height of the odontoid process of the axis and the maximum length of the femur were measured and statistically analyzed.

Results

The mean values for the height of the dens were 19.13±2.74 mm and 16.83±2.45 mm concerning the male and female dry skeletons respectively. The mean maximum length of the right femur bone was 43.04±2.32 cm for male and 39.90±2.40 cm for female skeletons. Data analysis revealed a statistically significant correlation (r=0.709, p <0.001) between the height of the odontoid process and the maximum length of the femur bone. A linear regression model expressing this association was created: Femur max length (in cm) = 32.874 + 0.531 x Dens height (in mm).

Conclusion

We present a new mathematical equation correlating one of the most studied long bones of the skeleton, the femur, with another "long" part of the bony structure of the human body- the C2 odontoid process.

## Introduction

The odontoid process, a term derived from the Greek word "odontas" (Greek: οδόντας, the tooth) is a tooth-shaped process that projects superiorly from the C2 vertebral body and plays a critical role for the function of the craniocervical junction. Anatomically, the dens is divided in four parts: the tip, the body, the neck and the base [1,2]. The dens separates from the atlas and fuses with the body of the axis between the 6th to 7th week of embryonic life. Before fusion, the dens is connected to the body of the axis with a cartilaginous plate, named dentocentral synchondrosis. This synchondrosis structure could be considered as the inferior border of the dens [1,3]. Its mark could be detected on the anterior surface of the axis as a bony crest above and parallel to the level passing through the inferior margin of the superior articular facets. Moreover, it can be depicted in adults by magnetic resonance imaging [4].

The odontoid process constitutes an important structure of the human body with unique characteristics. Studies in morphology and quantitative anatomy of the axis dens enjoy great interest among medical practitioners. However, no previous research has correlated dens dimensions with any long bone dimensions. On the other hand, long bones such as the femoral have been a common subject of study, with frequent finding correlations between them and body length and height. The presence of a relationship between the length of the dens and the length of the femur might be of great importance, embryological and clinical as well. Such an association between the two bones may also conceal a further relation with body length and height.

 Thus, the purpose of our study was threefold:

1) To study the height of the C2 dens in a number of Caucasian specimens and highlight the possible differences between males and females.

2) Accordingly, to study the length of the femur in a number of Caucasian specimens and compare between males and females.

3) To examine the presence of a possible relationship between the height of the C2 dens and the maximum length of the femur bone.

 To the best of our knowledge, there is no other study of this subject in the literature.

## Materials and methods

Forty-five dry adult skeletons were thoroughly examined, of which twenty-three were male (51,1%) and twenty-two were female (48.9%). The study specimens (mean age 75.5 years, SD 7.1), all of Caucasian (Hellenic) origin, were retrieved from cemeteries located in several Greek regions, including Pelloponisos, Thessalia, Crete and Thrace. Sex definition for each specimen was recovered by the cemetery records. All specimens with evidence of degenerative bone lesions, obvious skeletal pathology (tumor, fractures, osteoarthritis) and damages by taphonomic processes were excluded from the study. Specimen inspection and examination was authorized by the Research Ethics Committee of the Democritus University of Thrace and the local Municipal Authorities.

**Figure 1:**
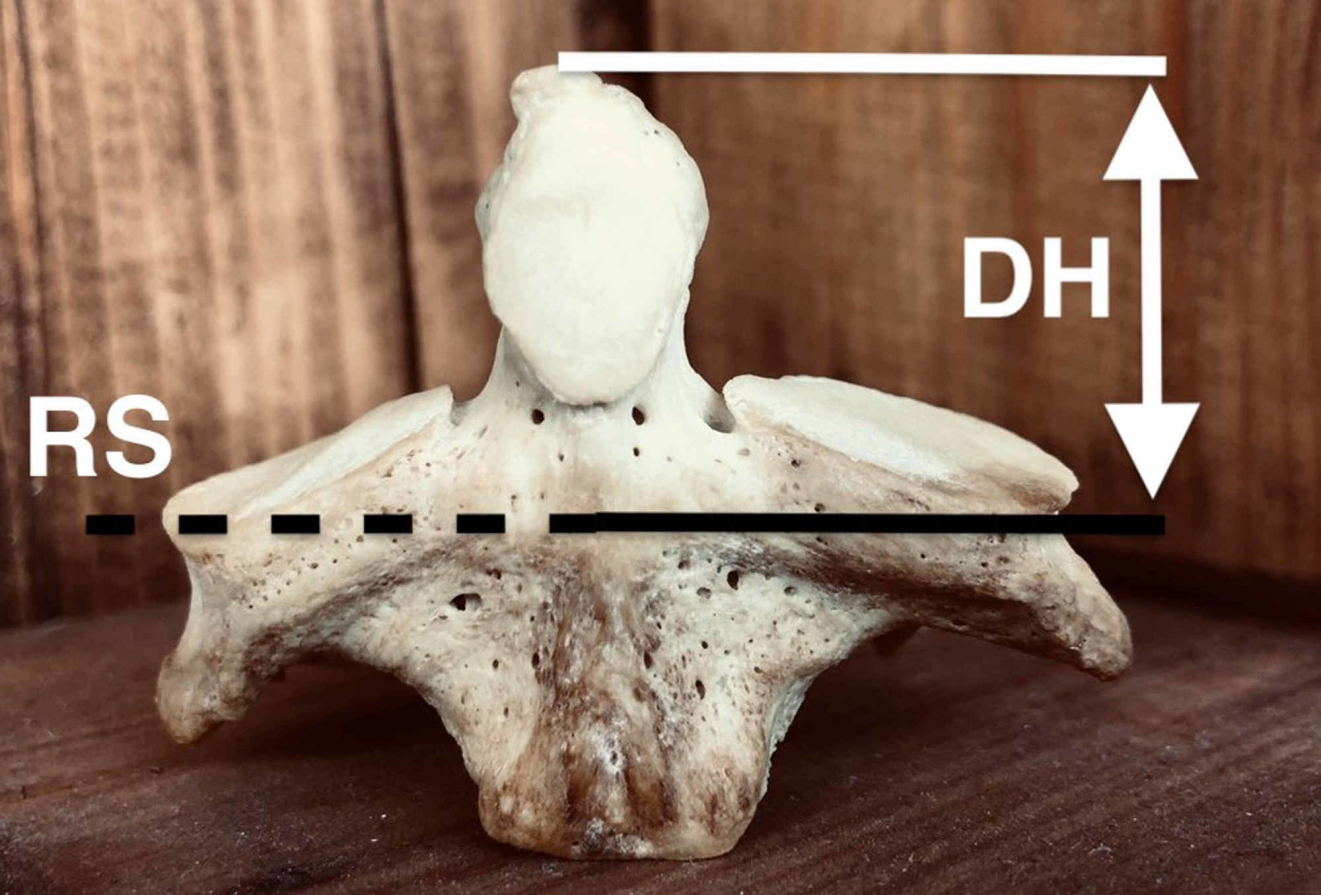
The axis vertebra DH: Dens Height, RS: Remnant of dentocentral synchondrosis.

**Figure 2:**
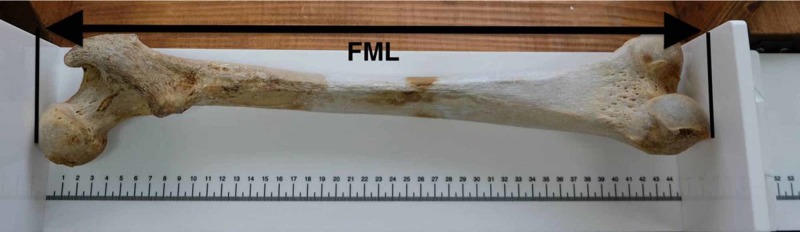
The right femoral bone FML: Femur Maximum Length.

Dens height and maximum femoral length were measured for each skeleton. A digital caliper with an accuracy of 0.01 cm was used for the height of the odontoid process, which was measured from its tip to the remnant of the dentocentral synchondrosis, below the level of the plane passing through the superior articular facets as indicated by the study of Cokluk et al. and Akobo S et al. (figure 1) [1,4]. An osteometric board with a linear scale of 60 cm in 1mm increment was used for the femur maximum length measurement. The vertical planes of the board were adjusted to the most proximal point on the femoral head and to the most distal point of the medial femoral condyle, with the femur placed on the board on its anterior surface (figure 2). The measurement of the right femoral length was used as a standard for the extraction of our equation.

 

The measurements were repeated by two different, independent researchers for the accuracy of the results to be ensured. Data analysis for inter-observer error showed a mean difference between the first and second measurement of -0.5 mm, indicating that there is no relevant difference between the two series of measurements.

 Statistical analysis of the data was performed by using the Statistical Package for the Social Sciences (SPSS), version 19.0 (IBM). The distribution normality of quantitative variables was tested with Kolmogorov-Smirnov test. All quantitative variables were expressed as the mean±standard deviation (SD). Student’s t test was used to determine differences in femur maximum length and dens length between males and females. The Pearson’s r correlation coefficient was used to assess the linear association between femur maximum length and dens length. Least square regression models were constructed in order to predict femur maximum length from the measurement of dens length males, females and all specimens. Coefficient of determination (R^2^) was calculated as a measure of the accuracy of these models. All tests were two tailed and statistical significance was considered for p values less than 0.05.

## Results

The mean, range and standard deviation of each parameter was calculated for male and female skeletons separately and for all 45 specimens (Table 1). Among the entire sample, the height of the dens varied from 12.51 to 22.17 mm with a mean value of 18.09 ± 2.83 mm. Regarding sex, the height of the dens varied from 13.10 to 24.49 mm for the male and from 12.51 to 22.17 mm for the female. The mean dens height was significantly greater in males compared to females (19.13 ± 2.74 vs 16.83 ± 2.45 mm, p=0.002) by 13.7% (mean difference, 2.30 mm, 95% CI=0.89 - 3.72 mm) (Table 1). The maximum length of the femur varied from 35.80 to 47.50 cm with a mean value of 41.61 ± 2.82 cm. Regarding sex, the maximum length of the femur varied from 39.10 to 47.50 cm for the male and from 35.80 to 43.60 cm for the female; the mean maximum length of the femur was statistically significantly greater in male compared to female (43.04 ± 2.32 vs 39.90 ± 2.40 cm, p<0.001) by 7.9% (mean difference, 3.14 cm, 95% CI=1.87 - 4.43 cm) (Table 1). Correlation analysis, using Pearson’s "r" correlation coefficient, showed statistically significant positive correlation between femur maximum length and dens length among males (r=0.628, p<0.001), females (r=0.647, p<0.001) and all specimens (r=0.709, p<0.001) (Table 2). 

**Table 1:** Dens height and femur maximum length values among all specimens and in relation to gender * statistical significance for the comparisons between males and females (Student’s t test)

		Total	Males	Females	P value*
Dens Height (mm)	Mean (SD)	18.09 (2.83)	19.13 (2.74)	16.83 (2.45)	0.002
	Range	12.51 – 24.49	13.10 – 24.49	12.51 – 22.17	
Femur maximum length (cm)	Mean (SD)	41.61 (2.82)	43.04 (2.32)	39.90 (2.40)	<0.001
	Range	35.80 – 47.50	39.10 – 47.50	35.80 – 43.60	

**Table 2:** Association between femur maximum length and dens length Association between femur maximum length and dens length expressed as Pearson’s r correlation coefficient (statistical significance is also given) among all specimens, males and females

	All specimens	Males	Females
Pearson’s r correlation coefficient	0.709	0.628	0.647
P value	<0.001	<0.001	<0.001

Linear regression models, in which the femur maximum length (in cm) was considered as the dependent variable and the dens height (in mm) was considered as the independent variable were obtained. Since there were established sex differences, the following two models for each sex were created:

Male:

Femur m = 32.874 + 0.531 x Dens h

Female:

Femur m = 29.221 + 0.634 x Dens h

Femur m = Right femur max length (in cm)

Dens h = Dens height (in mm)

A linear regression model based in measurements from all specimens regardless of sex, that can be used in cases of unknown sex is the following:

Femur m = 28.862 + 0.705 x Dens h

The resulting models can provide an estimation of the femur maximum length from the measurement of dens length. Coefficient of determination indicates that dens height was a strong determinant for the femur maximum length among all specimens (R^2^=50.2%), males (R^2^=39.4%) or females (R^2^=41.9%).

## Discussion

There are various methods for measuring the height of the odontoid process of the axis vertebra. Some researchers note that it may be measured from its tip to the level of a plane through the superior articular facets, while others support that it can be calculated with an established mathematical relationship; Dens height = overall dens height- height to base of the dens 5-[7]. However, to achieve maximum anatomical precision, we have decided to measure the dens height from its tip to its base, as defined by the embryologic remnant of the C1-2 intervertebral disc, a cartilaginous plate that may remain until old age [1]. This line of fusion between dens base and the body of the axis, the dentocentral synchondrosis, lies below the level of the plane that passes through the axis’ superior articular facets [4] (Figure 1).

For the measurements of the right femoral maximum length, a commonly used method was chosen, with the femur placed on the osteometric board on its anterior surface. The distance between the most proximal point of the femoral head and the most distal point of the median femoral condyle represented the maximum length of the bone [8-10] (Figure 2). There are studies indicating no statistical difference between the left and right femoral length, while others support the opposite [11-14]. It seems though, that early anthropological researchers presumed that there were significant length differences between the right and left femurs in man [15]. Studies noted that all long bones of the right side of the human body are longer than those of the left, with the exception of the left femur [16]. The left femur is not only longer but it also presents a variability in weight, having a greater correlation with the total skeleton weight [17]. For the extraction of our equation, we have chosen to measure the right femoral length, as De Mendonça did in his study [9].

The results derived from the present study confirmed the original idea; a statistically strong relationship exists between the height of the dens and the maximum length of the femur. Three equations, two for both sexes and one for undetermined sex (insufficient or damaged remains) were produced. It became obvious that if the femur maximum length increases, the dens height increases too and vice versa. It should be noted that no similar data exist, to our knowledge, to the available literature.

In our study, we proved a direct analogy (ratio) between the two measurements, as accurate as possible and independent from absolute values, which vary greatly between subjects’ races, sex, age, nutritional habits, genetics and other parameters. Geometrical studies of discrete anatomical formations, including determination of dimensions, volume, shapes, axons, diameters, inclinations and angles, are innumerable in the international literature and their results have induced the emergence of multiple applications to a wide range of clinical and surgical disciplines. Usage of a clear, quantifiable and feasible osteometric index not only broadens anatomical knowledge, but it could also be an invaluable implement to the assessment of skeletal deformities or the estimation of acral growth retardation and other developmental disorders.

In regard to the studies correlating femoral and other long bones length with the stature of the human body, which date from over a century, we agree that a large sample of young and healthy individuals should be used to certify the accuracy of such a formula, and still it will be dependent on the population from which it was derived [18-19]. Iin his study, De Mendonca produced a formula, using simple linear regression which may give an estimation of the body height by conducting measurements of the long bones [9]. A study from Schaffner et al. suggested that there was no significant correlation between dens dimensions and body height and weight [5]. However, body height is not a constant and reliable parameter, as it can be affected and altered during life by several factors, including age, pathological conditions, nutrition, professional context, level of activity etc.

The exact dens height is of crucial importance in the neurosurgical treatment of its fractures. Fractures of the C2 odontoid process are the most common injuries of the upper cervical spine. Anterior odontoid osteosynthesis or posterior cervical fusion with or without screw fixation are surgical techniques requiring detailed determination of the dens length, which is not always possible when it is deformed and/or displaced. Our equation may assist with the calculation of the odontoid process height and therefore the screw’s size, through the measurement of the length of the right femoral bone [20-22].

The presented formula will certainly draw the interest of scientists involved in anthropological studies. Human remains from archaeological sites could present a real challenge for osteoarchaeologists, especially when they include funerary monuments with multiple burials. Application of a simple formula for distinguishing and matching skeleton parts with precision could have a great impact on saving time and adding scientific accuracy to the processing of bones and the reconstruction of discrete skeletons. 

Such formulae, like the one suggested in the present study, could also be applicable in forensic medical practice. Bone biological samples encountered in routine forensic casework are often highly degraded or limited in quantity. It can be extremely difficult to obtain sufficient DNA for analysis in cases of burnt, charred or otherwise damaged bones [23]. Thus, an equation which correlates the height of the axis dens with the maximum length of the femoral bone may facilitate matching of human remains, mainly after major catastrophes or accidents.

## Conclusions

The length of the femoral bone has been used by various researchers in human height measurement calculations, combined sometimes with comparisons to other bones, usually the long ones. This is the first study which connects the femoral length and the odontoid process with an equation which may be used in clinical anatomy, anthropology and forensics. A statistically high correlation between the maximum length of the femur and the height of the odontoid process was observed. Both are being bound to each other by a mathematical formula introduced in this study. Such formulae enrich the known measurement studies in anatomy and could provide insight into surgical, forensic and anthropological procedures.
